# Meningitis Serogroup W135 Outbreak, Burkina Faso, 2002

**DOI:** 10.3201/eid1306.060940

**Published:** 2007-06

**Authors:** Nicolas Nathan, Angela M.C. Rose, Dominique Legros, Sylvestre R.M. Tiendrebeogo, Catherine Bachy, Egil Bjørløw, Peter Firmenich, Philippe J. Guerin, Dominique A. Caugant

**Affiliations:** * Epicentre, Paris, France; † Ministry of Health, Ouagadougou, Burkina Faso; ‡ European Programme for Intervention Epidemiology Training, Oslo, Norway; § Norwegian Institute of Public Health, Oslo, Norway; ¶ Médecins sans Frontières, Bereldange, Luxembourg; # World Health Organization Collaborating Centre for Reference and Research on Meningococci, Oslo, Norway; 1 Deceased.

**Keywords:** Burkina Faso, children, epidemiology, Neisseria meningitidis serogroup W135, outbreak, sensitivity, specificity, dispatch

## Abstract

In 2002, the largest epidemic of *Neisseria meningitidis* serogroup W135 occurred in Burkina Faso. The highest attack rate was in children <5 years of age. We describe cases from 1 district and evaluate the performance of the Pastorex test, which had good sensitivity (84%) and specificity (89%) compared with culture or PCR.

Meningococcal epidemics in sub-Saharan Africa have been caused, until recently, mainly by *Neisseria meningitidis* serogroup A (*1*); strains of serogroup W135 have been isolated sporadically (*2*). In 2000 and 2001, serogroup W135 was associated with outbreaks in pilgrims to Mecca, Saudi Arabia, followed by several clusters of cases worldwide (*3*–*5*).

Laboratory confirmation of meningococcal meningitis is conducted by using antigen detection in cerebrospinal fluid (CSF), culture, or PCR techniques (*6*,*7*). The Pastorex latex agglutination test (Bio-Rad Laboratories, Marnes-La-Coquette, France) is the most common rapid test used in the field to detect *N*. *meningitidis* serogroup W135 antigen, although it cannot differentiate serogroups W135 and Y.

In January 2002, a preventive mass-vaccination campaign with a bivalent A-C polysaccharide vaccine started in Burkina Faso in districts with low vaccine coverage in 2001. In the week of January 28, 2002, Pama District crossed the epidemic threshold of 10 cases/100,000 per week (*8*). This district had achieved 100% vaccination coverage of the target population (2–29 years of age) in 2001 (Médecins sans Frontières internal report). Four weeks later, 4 other districts that had achieved ≥80% vaccination coverage in 2001 (Epicentre internal comm.) crossed the epidemic threshold (*8*). By mid-March 2002, the World Health Organization (WHO) Collaborating Centre for Reference and Research on Meningococci (CCRRM) in Oslo, Norway, confirmed most of these cases as caused by serogroup W135. Because the A-C vaccine could not provide protection against serogroup W135, the Ministry of Health ended the vaccination campaign.

The epidemic in Pissy District (population 520,314 in 2002) was investigated by the Burkina Faso Ministry of Health and WHO. We evaluated the Pastorex test for detecting *N*. *meningitidis* serogroup W135 in patients at Pissy Medical Health Centre (MHC).

## The Study

A suspected case was defined as a febrile syndrome of sudden onset, associated with headache, stiff neck, or vomiting. A probable case was any suspected case with either a positive or doubtful result on direct microscopic examination of CSF. A confirmed case was a probable case with serogroup identification in CSF by culture, Pastorex test, or PCR. Patients with suspected cases were hospitalized and treated with a suspension of chloramphenicol in oil or another antimicrobial drug, as appropriate (*9*). Patients with severe cases were routinely transferred to Yalgado Ouédraogo National Hospital in Ouagadougou. Attack rates by age group were calculated for cases reported during weeks 6–18 (February 4–May 5) by using population data for Pissy District and standard age-group distributions for developing countries (*10*).

CSF samples from patients with suspected cases during weeks 17–20 (April 21–May 15) were examined at Pissy MHC by direct macroscopic and microscopic techniques, including Gram stain and leukocyte counts (as long as the CSF was not bloody). Pastorex rapid agglutination test was also used following the manufacturer’s instructions.

A positive result for direct microscopic examination was indicated by numerous organisms or >10 leukocytes/mm^3^ CSF. A doubtful result was indicated by a rare organism and <10 leukocytes/mm^3^ CSF (or count not made). Any other result was considered negative. If results of direct microscopy were positive or doubtful, the remaining CSF sample was placed in 2 bottles of trans-isolate medium (provided by WHO CCRRM in Oslo). One bottle was sent for culture to the Charles de Gaulle Paediatric Hospital Laboratory in Ouagadougou. For quality control, the other was sent to WHO CCRRM for culture or PCR.

In Oslo, 100 μL of each CSF sample in trans-isolate medium was plated onto chocolate agar and chocolate agar containing 7.5 mg/L colimycin, 0.5 mg/L linocmycin, 1.0 mg/L amphotericin B, and 5.0 mg/L trimethoprim. Plates were incubated at 35°C in an atmosphere of 10% CO_2_ for <3 days, and meningococci were identified by standard methods (*11*). PCR was performed as previously described (*6*,*7*) on samples that were either contaminated or culture negative for meningococci.

Performance of the Pastorex test was measured by calculating sensitivity and specificity by using culture or PCR results from WHO CCCRM as the comparison standard. Samples with contaminated cultures and those that inhibited PCR (clinical specimens may contain inhibitory substances [*12*]) were considered negative, as were undetermined results. Positive and negative predictive values (PPV and NPV, respectively) were also calculated.

**Figure 1:**
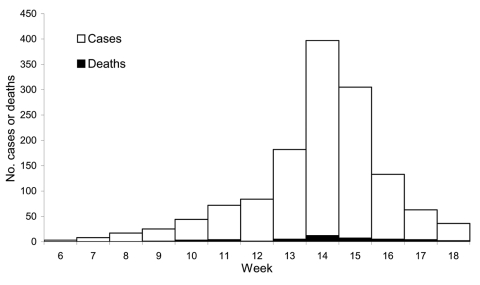
Number of meningitis cases (N = 1,325) and deaths reported during weeks 6–18 (February 4–May 5) at Pissy Medical Health Centre, Burkina Faso, 2002.

Of 2,130 patients with suspected cases reported in Pissy District during weeks 6–18, the conditions of 1,325 (65%) were diagnosed and treated at Pissy MHC (Figure 1); 44 died (case-fatality rate [CFR] 3%). Age was available for 1,307 (99%) of 1,325 patients. The highest attack rate was in patients <1 year of age (1,092/100,000), followed by patients 1–4 years of age (660/100,000). The attack rate continued to decrease with age (Table 1). Vaccination history was provided by 1,137 patients with suspected cases (86%), of whom 791 (70%) had been vaccinated against meningitis; information on year of vaccination was unknown.

**Table 1:** Attack rates per 100,000 population by age group for suspected meningitis cases, Pissy Medical Health Centre, Burkina Faso*

Age group, y	Population	No. cases	Attack rate/100,000
<1	19,772	216	1,092
1–4	68,681	453	660
5–14	145,688	289	198
15–29	145,688	220	151
>30	140,485	129	92
All	520,314	1,307	251

*Weeks 6–18 (Feb 4–May 5), 2002 (n = 1,307). Population data are for the entire district of Pissy.

Confirmed case-patients showed typical clinical features (*13*) (Table 2) and a CFR of 10%. Their ages ranged from 5 months to 19 years (median 4 years); the male:female ratio was 1.6:1. The 3 classic clinical signs of meningitis (headache, fever, and stiff neck) were present in 10 case-patients (33%).

**Table 2:** Characteristics of patients with confirmed cases of infection with *Neisseria meningitidis* W135, Pissy District, Burkina Faso, Apr–May 2002 (n = 31)

Characteristic	No. (%)
Age group, y	
<1	1 (3)
1–4	16 (52)
5–14	13 (42)
15–30	1 (3)
Male sex	19 (61)
Symptom onset, d*	
<1	2 (7)
1–2	18 (60)
3–4	10 (33)
Received antimicrobial drug†	25 (89)
Temperature, °C*	
<38	8 (27)
38.1–39.9	12 (40)
>40	10 (33)
Other clinical signs	
Headache	21 (70)
Vomiting	22 (73)
Anorexia	22 (73)
Stiff neck	16 (53)
Fever, stiff neck, and headache	10 (33)

*Data missing for 1 case-patient. †Data missing for 3 case-patients.

During weeks 17–20, successful lumbar punctures (LPs) were performed in 260 patients with suspected cases at Pissy MHC. Thirty-one were positive for meningitis serogroup W135 by culture, PCR, or Pastorex test. CSF was clear in 6 (19%) samples, cloudy in 22 (71%), and bloody in 3 (10%). Among 6 clear CSF samples, 3 had doubtful results by direct microscopy and were confirmed only by Pastorex test.

**Figure 2:**
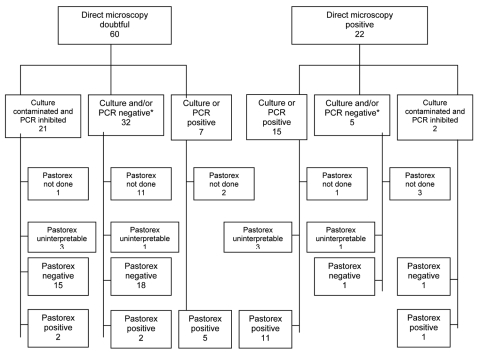
Schematic representation of results of culture or PCR performed on 82 cerebrospinal fluid samples with doubtful or positive direct microscopic results. See text for definitions of doubtful and positive direct microscopic results. *With the other result contaminated (culture) or inhibited (PCR).

Eighty-two CSF samples from all probable case-patients were sent to WHO CCCRM. These samples were tested by direct microscopy, and most were tested by Pastorex test in Burkina Faso. Sixty samples had doubtful results, and 22 had positive results by direct microscopy (Figure 2).

The Pastorex test on 64 samples tested by culture or PCR showed a sensitivity of 84% (95% confidence interval [CI] 60%–97%) and a specificity of 89% (95% CI 76%–96%) for detection of serogroup W135. PPV and NPV for this test were 76% (95% CI 53%–92%) and 93% (95% CI 81%–99%), respectively.

## Conclusions

The meningitis epidemic in Burkina Faso in 2002 was the largest reported outbreak caused by *N*. *meningitidis* serogroup W135 to date (*3*,*13*), with nearly 13,000 suspected cases (*9*). We report a portion of this epidemic at 1 health center, which represented ≈10% of suspected cases nationwide. Attack rate was highest in patients <5 years of age and decreased with age. Symptoms and CFR of confirmed case-patients were typical for meningitis. The Pastorex test had adequate sensitivity (84%) and specificity (89%) for detecting the W135 serogroup, similar to those found under ideal laboratory conditions (85% and 97%, respectively [*14*]).

An effective public awareness campaign and fear in the population (because of lack of suitable vaccine) resulted in large numbers of patients with suspected cases arriving at health centers throughout the country, and more LPs were conducted than expected. This situation—and the case definition, which was sensitive but not specific—explained why of 260 LPs performed in a 4-week period at the end of the epidemic, only 31 were positive. Routine transfer of severe case-patients from Pissy MHC to the national hospital explained the lower CFR reported from Pissy MHC (3%) than for the whole epidemic (12%; [*9*]).

During this study, 25% of CSF samples analyzed with Pastorex test were unreadable, which may have been caused by differences in the serogroup W135/Y reaction in this test. In addition, difficulties in reading this test (possibly because of a lack of expertise in reading agglutination test results) have been reported in the field during epidemics.

The Pastorex test provides faster results than either culture or PCR (minutes vs. days) and requires less training and no specialized equipment other than a refrigerator, centrifuge, and water bath. It is thus more appropriate for developing countries with limited resources (*15*), despite relatively high costs (in 2005 kits cost ≈€11 per CSF sample analyzed). The high NPV of this test and its rapidity make it an important case management tool because cases of nonmeningococcal meningitis during an outbreak require different treatment. Other studies have shown this test to have high sensitivity and specificity under ideal conditions for both serogroups A (*14*,*15*) and W135 (*14*). Further study is needed to confirm the validity of this test under epidemic conditions in the field, particularly readability of results for serogroup W135.
